# Emerging Perspectives in Anti-aging Treatments Using Anti-Fibrotic Strategies

**DOI:** 10.14336/AD.2025.1204

**Published:** 2026-10-17

**Authors:** Yu Wang, Chenshi Xi, Jiangli Yang, Mingye Wang, Linghui Zeng, Lingxiao Zhang, Chong Gao

**Affiliations:** ^1^Brain and Cognition Research Institute, School of Medicine, Hangzhou City University, Hangzhou 310015, China.; ^2^Hangzhou Institute for Advanced Study, University of Chinese Academy of Sciences, Hangzhou 310024, China.; ^3^Interdisciplinary Nanoscience Center, Aarhus University, Aarhus C 8000, Denmark.

**Keywords:** fibrosis, aging, cancer, chinese medicine, gene and cell therapy, nanomedicine, organoids

## Abstract

Aging involves chronic organ degeneration, characterized by a continued decline in cellular regenerative ability and excessive buildup of extracellular matrix (ECM), leading to organ fibrosis. Fibrosis is now widely recognized as a key sign of organ failure, which has inspired new anti-aging strategies through anti-fibrosis treatments. In cancer, excessive ECM around and within tumors, known as desmoplasia, helps support tumor growth and malignancy. Age-related chronic inflammation and impaired tissue regeneration lead to a range of cell changes, which favor the development of fibroblast-like types, promote unchecked ECM buildup, and increased tissue stiffness. Notably, many mechanisms of aging closely overlap with those behind fibrotic progression. Understanding the critical cell groups, especially cancer-associated fibroblasts, could open promising options for anti-fibrotic and cancer treatments. The integration of progress in molecular medicine, traditional herbal therapies, and new technologies provides a powerful path for drug discovery and therapy development. In this review, we outline the main cell types and molecular pathways involved in organ aging and fibrosis. We also highlight the recent advances in anti-fibrotic Traditional Chinese Medicine (TCM) and gene and cell therapies in cancer and anti-aging research. Lastly, we examine the role of new technologies, including nanomedicine and organoid models, in the development and testing of drugs for anti-fibrotic therapies.

## 1. Introduction

Growing interest in anti-fibrotic therapy reflects increasing recognition of fibrosis as a major contributor to tissue dysfunction and aging. Fibrosis, driven by the deposition of extracellular matrix (ECM) deposition, disrupts tissue homeostasis, impairs regeneration, and promotes age-related decline [1]. The imbalance between tissue regeneration and ECM overproduction leads to irreversible tissue stiffness, chronic degeneration, and organ failure [1]. As a global healthcare burden, the discovery of key anti-fibrotic targets paves the way for new treatments for diseases and longevity management. Aging is closely associated with fibrotic remodeling across multiple tissues [2]. Fibrotic conditions, such as idiopathic pulmonary fibrosis (IPF), liver fibrosis, chronic kidney fibrosis, and skeletal muscle fibrosis, are increasingly recognized as contributing to aging-related organ functional decline [3]. Key signaling molecules, such as transforming growth factor-beta (TGF-β) and connective tissue growth factor (CTGF), are also secreted from senescent cells or drive aging-related biological processes, including inflammatory responses, senescence, DNA damage, and oxidative stress [4, 5].

Fibroblasts are the primary functional cells of connective tissue, responsible for maintaining the ECM across tissues and organs. In an ideal scenario, lost tissue that is replaced by new cells of the same type and function could restore the original organ's structure and function. When regeneration is incomplete, the tissue repair mechanisms initiate the deposition of ECM and form a scar, providing structural support to close the wound [6]. Fibrosis represents the pathological transition from transient to persistent scarring, reflecting both cellular plasticity and microenvironmental alteration [4]. During fibrosis, interactions between fibroblasts and other cells results in accelerated ECM deposition and tissue degradation. The alteration in various tissue cells, including immune cells, epithelial cells, cardiomyocytes, and myoblasts, initiates a cascade of changes in ligand signaling secretion, cell mechanical environment, and cell lineage transitions, all of which contribute to the stiffness of the ECM.

Efforts to address fibrosis are ongoing. Pirfenidone, nintedanib, and resmetirom have been approved as anti-fibrotic drugs for treating idiopathic pulmonary fibrosis (IPF), systemic sclerosis-associated interstitial lung disease (SSc-ILD), as well as metabolic dysfunction-associated steatohepatitis (MASH) [2, 7]. Due to the challenges in uncovering the complex mechanism of fibrosis, many anti-fibrotic reagents have failed to demonstrate clinical benefits. Recently, advanced medical methods, including alternative therapies and cell and gene therapies, have offered promising approaches for anti-fibrosis. These new methods, such as traditional Chinese Medicine (TCM), anti-fibrotic chimeric antigen receptor (CAR) T cells or NK cells, antibody-drug conjugates (ADCs), nanomedicine, siRNA, aptamers, and antisense oligonucleotides (ASOs), are showing their innovation in preclinical or clinical studies.

**Figure 1.**
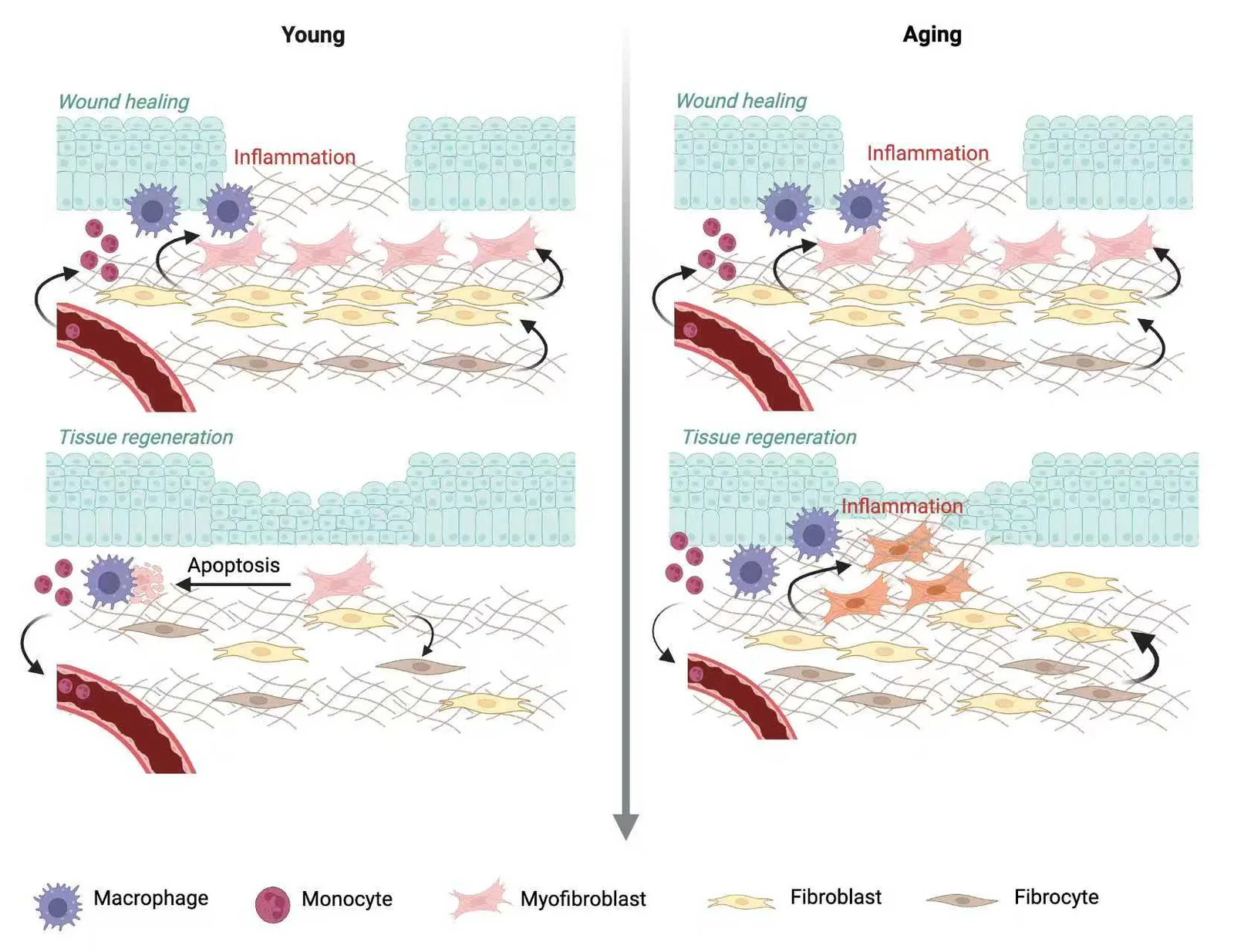
**Aging accelerates tissue fibrosis during regeneration**. Appearance and crosstalk of pro-fibrotic cell types in the progress of tissue regeneration under young or aged circumstances. Aging disrupts this resolution process by delaying tissue regeneration and sustaining myofibroblast activation. Persistent myofibroblast activity leads to prolonged macrophage recruitment and chronic local inflammation. Simultaneously, fibroblast numbers remain elevated due to impaired repair and unresolved inflammatory signals. (This diagram was created with BioRender: https://www.biorender.com/)

Plenty of cell types are involved in the progression of aging-associated diseases, including organ dysfunction and cancer development. During pathological development, these cells undergo alterations in their expression profiles, thereby displaying a lineage transition. Fibrosis plays a pivotal role in aging-related diseases, not only by altering the physicochemical microenvironment for cellular behaviors within tissues, but also by driving pathological changes through pro-fibrotic processes and associated alterations in cellular composition. All these changes are associated with the key cell types and the regulatory signaling involved in aging-associated fibrosis. In this review, we summarize the key cell types that participate in physiological tissue repair and pathological fibrosis, as well as the unique contributions of these cells associated with aging. We then introduced the recent advantages of new medication methods in anti-fibrotic therapy. Ultimately, we further discussed new technologies, including nanomedicine and organoids, that can accelerate the development of anti-fibrosis drugs.

## 2. Cross-cell network of aging-associated fibrosis

Following tissue impairments, a series of homeostatic reparative processes is initiated to restore organ function and structure, forming the early stage of fibrosis. The scarring process is triggered after the injury. Fibrosis is thought of as the shift in ECM production, where normal tissue remodeling becomes dysregulated, resulting in excessive ECM deposition. ECM, at the physiological level, acts as a scaffold for the migration of reparative cells into the injury region, facilitating tissue regeneration. However, under pathological conditions, over-secretion of fibrotic factors, including TGF-β and connective tissue growth factors, activates the local fibroblasts and macrophages, leading to the formation of fibrosis. In most organ dysfunctions, continuing tissue degeneration and dysregulated ECM deposition are trapped in a vicious circle. Recent studies uncovered the cell crosstalk that drives fibrosis and provide the targets for anti-fibrotic therapy in specific organs. Pro-fibrotic cells contribute to the fibrosis program via direct production of the ECM or interacting with ECM-producing cells or both. Some of these cells exert the function of ECM maintenance or tissue regeneration and displayed dysregulation during fibrosis. While other cell types, such as myofibroblasts, are most likely to transdifferentiate from different cell types in the progression of fibrosis. In this section, we outline the key cell types with pro-fibrotic roles and their functions in aging-related tissue fibrosis, as well as their special roles in cancer progression (Fig. 1).

### 2.1 Fibroblasts

Fibroblasts play a central role in the production of ECM and lead to fibrosis pathology. As the principal cell type of connective tissue, fibroblasts exhibit considerable heterogeneity across different organs [8]. Moreover, the coexistence of various populations of fibroblasts can be observed in the same organs [9]. Research on single-cell RNA sequencing focuses on clarifying the intra-organ and inter-organ heterogeneity of fibroblasts [9]. Diversity of the fibroblasts, such as dermal fibroblasts in the skin, intestinal fibroblasts, or portal fibroblasts in the liver, displays the tissue-specific features and maintains the structural integrity by ECM production [10, 11]. During aging, the number of fibroblasts in dermal tissue decreased significantly. It was demonstrated that aging-associated fibroblast senescence underwent a state of cell cycle arrest, which, on the other hand, promoted fibrosis and inflammation [12]. Aging affects fibroblasts in multiple aspects, primarily through changes in stress response, lineage commitment, and expression heterogeneity. Increased susceptibility to fibrosis occurs during aging in various tissues, which may underline the altered interaction between fibroblasts and ECM or tissue cells. Senescent fibroblasts undergo cell cycle arrest and become metabolically inactive, leading to an increased fibrotic environment and impaired wound healing [13]. Activating metabolic fibroblasts increased the production of profibrotic factors, like TGF-β1 [14]. Such aging-associated phenotypes in fibroblasts are reported in most profibrotic cells, known as the senescence-associated secretory phenotype (SASP), which is characterized by the secretion of cytokines, chemokines, growth factors, and proteases by senescent cells [15]. Signaling pathways, such as TGF-β1, Wnt, Yap/TAX, and matrix metalloproteinases (MMPs), further contribute to the dysfunctions of the ECM, which plays a crucial role in promoting the aging-associated fibrosis [16]. This change of the ECM, including the accumulation of fragmented collagens, oxidation, glycation, and protein aggregates, resulted in the stiffness and altered cellular niches of the tissue [17, 18]. The initial protumor mechanism of SASP depends on its role in promoting tumor cell proliferation. It was reported that senescent fibroblasts can trigger the hyperproliferation and progression of preneoplastic epithelial cells, which further accelerates the tumorigenesis [19].

Fibrocytes, which are recognized as fibroblasts in the quiescent stage, display a morphologically smaller size. During aging, circulating fibrocytes exhibit lineage commitment to myofibroblasts, which may contribute to age-dependent fibrosis [20]. Fibrocytes from older individuals exhibit an enhanced ability to differentiate into myofibroblasts, which secrete collagen and contribute to the development of fibrosis [20]. The transition of fibrocytes to fibroblasts is initiated by key factors associated with aging, such as TGF-β1 [21]. Beyond the dysregulated activity of fibroblasts, multiple tissue-specific cell types undergo lineage transitions toward fibroblast-like states, contributing to fibrotic remodeling. This process amplifies ECM accumulation and accelerates the progressive loss of standard tissue architecture and function.

### 2.2 Cancer-associated fibroblasts

The progression of cancer heavily depends on the support to maintain continuous tumor growth and metastasis. In contrast, normal fibroblasts surrounding the tumor were unable to hinder the invasion and metastasis of cancer cells [22]. Cancer-associated fibroblasts (CAFs) are a special population of activated fibroblasts found in the tumor microenvironment (TME) [23, 24]. CAFs are derived from multiple sources, including tissue-resident fibroblasts, stellate cells (in organs such as the liver and pancreas), bone marrow-derived mesenchymal stem cells, pericytes, adipocytes, endothelial cells, and epithelial cells. Based on their characteristics, CAF subtypes can be identified by specific markers, such as myofibroblast-like CAFs, inflammatory CAFs, and antigen-presenting CAFs, which may play distinct roles in TME regulation [25, 26]. Recent advances in single-cell omics offer opportunities to further elucidate the heterogeneity of CAF in a context-dependent manner [27]. Aging could induce an altered phenotype of CAFs. Senescent CAFs (senCAFs) exhibit increasing aging markers, secrete pro-inflammatory factors and ECM components, which were indicated to promote the growth, immunosuppression, and therapeutic resistance of tumors [28, 29]. SenCAFs promote ECM remodeling and tumor tissue stiffness, providing a therapeutic-resistance niche. It was reported that senCAFs increased the production of collagen and fibronectin, which further creates the physical barrier to promote cancer cell invasion, metastasis and chemoresistance [22].

Metabolic activity of senescent cells represents the secretion of cytokines, chemokines, growth factors, etc., named as the senescence-associated secretory phenotype (SASP). It is reported that senCAFs are the main types of senescent cells in the tissue of non-small cell lung cancer (NSCLC) [30]. Senescent CAFs exert complex pro- and anti-tumorigenic functions. They could alter the composition of the ECM, which provides a niche support to tumor growth and invasion. Ye et al. discovered a subset of myCAF that is senCAF in mouse and human breast tumors. It is shown that these myCAFs restrict NK cell-mediated killing and thereby contributing to the progression of breast cancer [31]. A key role of senCAF in tumor regulation suggests it is a potential target for anti-cancer therapy. Assouline et al. reported that depletion of senescent CAFs using the genetic approach or the Bcl-2 inhibitor ABT-199 (venetoclax) could increase the proportion of activated CD8^+^ T cells in mouse pancreatic carcinomas, while induction of CAF senescence had the opposite effect [32]. In hepatocellular carcinoma, senCAFs are strongly correlated with cancer stemness, and CTHRC1 is identified as a novel prognostic and therapeutic biomarker to predict poor prognosis that enriched in senCAFs [33].

### 2.3 Epithelial cells

Epithelial cells also play a critical role in a series of biological events, including injury response, epithelial-mesenchymal transition (EMT), production of fibrotic factors, and interactions with immune cells [34]. The wound response triggers a series of cellular events in epithelial cells, including cell adhesion, migration, proliferation, and differentiation, which collectively facilitate the tissue repair process [35]. Aging epithelial cells play a significant role in the development of fibrosis, particularly in the context of tissue scarring. Take idiopathic pulmonary fibrosis (IPF) as an example; alveolar epithelial cells undergo senescence during aging and exhibit the SASP feature [36]. SASP of epithelial cells then stimulates fibroblasts and differentiates into myofibroblasts, which continuously secrete ECM and contribute to the fibrosis [3]. A previous study reported that aging induces cell cycle arrest in epithelial cells, resulting in an injury response that produces excessive proliferative growth factors, leading to myofibroblast activation and fibrosis [35]. This crosstalk of EpMT is increasingly recognized as the key mechanism contributing to fibrosis and organ dysfunction [36]. Aging is highly associated with increased EpMT in various tissues. Emerging evidence indicates that aging-induced factors, including ANGPTL4, interleukin-11, and SIRT1, play essential roles in EpMT [37-39].

Epithelial and endothelial cells, which act as the covering layer, are susceptible to tissue injury. Endothelial cells share the mechanisms with epithelial cells in contributing to the aging-associated tissue fibrosis. For skin fibrosis, it was demonstrated that the apoptosis of vascular endothelial cells was first detected during the progression of the fibrosis change [40]. Marks of endothelial-mesenchymal transition (EnMT) were detected in skin fibrosis following the microvascular injury. This pathological change exposes the endothelial basement membrane. It induces a series of downstream changes, such as platelet activation, which causes the release of profibrotic factors, including platelet-derived growth factor (PDGF), serotonin, and TGF-β [41-43]. The defects in the clearance of dead cells trigger a profibrotic and proinflammatory mechanism, thereby inducing the chronic accumulation of ECM and tissue fibrosis.

EpMT/EnMT also plays a critical role in tumor development. During tumor development, EMT provides the cell transition niche that allows epithelial cells to develop into the myofibroblast lineage [44]. Senescent epithelial cells established a maladaptive process and displayed SASPs to promote chronic fibrosis and cancer progression. Ionizing radiation therapy not only induces the cell death of cancer cells but also results in senescence. Radiation-induced lung fibrosis (RILF) has been shown to reduce the survival rate of cancer patients markedly [45]. Such injury circumstances promote the EpMT/EnMT process. For RILF, alveolar type II epithelial (AT2) cells serve as the primary targets in the EpMT/EnMT, which are highly relevant in clinical search for effective targets [46].

### 2.4 Myofibroblasts

Myofibroblasts are a specialized cell type that plays a critical role in the progression of fibrosis. It was identified with the ability to contract and produce ECM, and was considered to promote the dysfunctional changes in multiple organs [47]. In the condition of tissue injury, myofibroblast activation would be switched on by producing collagen-rich ECM and engaging the newly formed ECM into mechanically stable scar tissue [47, 48]. Formation of myofibroblasts is tightly associated with tissue repair stages. During hemostasis, platelets initiate bleeding stop and form the fibrin clot, which also serves as a scaffold for growth factors to recruit cells that populate the early wound bed. Following the inflammatory stage to fight infections, the proliferation phase is featured by the recruitment of repair macrophages and migration of adjacent normal tissue to divide and migrate into the wound. Afterward, in the tissue remodeling stage of wound healing, epithelium regenerates, and fibroblasts produce collagen. Myofibroblasts may form during the tissue remodeling stage, and they could be cleared in physiological repair by apoptosis, which allows scarless healing [49].

Myofibroblasts are marked with cytoskeletal actin-myosin bundles. Such structure was named as stress-fibers *in vitro*, which are the prerequisite underpinning for their contractile functions [49]. Stress fibers of myofibroblasts may facilitate the proliferation, migration, and ECM production, aiming at the accelerated progress of tissue repair. A series of biological mechanisms contributes to the activation of myofibroblasts. At tissue repair conditions, a stiffening environment during ECM production may change the biomechanical niche, triggering signaling pathways involving YAP/TAZ [50].

In aging subjects, myofibroblasts display reduced contractibility, thereby altering the mechanosensing pathway and cell-matrix interactions. Senescent myofibroblasts are marked by impaired dedifferentiation and apoptosis resistance. On the other hand, age-related tissue stresses, such as oxidative stress, endoplasmic reticulum (ER) stress, and changes in ECM composition, would further promote the differentiation of myofibroblasts [51, 52]. Increased differentiation and decreased apoptosis of myofibroblasts form the vicious cycle that leads to fibrosis and tissue dysfunction. It was reported that AMPK signaling could inhibit myofibroblast differentiation by suppressing the TGF-β, NF-κB, STAT3, and YAP/TAZ pathways, which contribute to aging-related cardiac and pulmonary fibrosis [53]. Sosulski et al. reported that autophagy was diminished, associated with the level of oxidized proteins and lipofuscin in response to lung injury in aging mice. The reduction of autophagy flux, which is mediated by TGF-β, may contribute to the differentiation of fibroblasts to myofibroblasts [54]. In myxomatous mitral valve disease, TGF-β/PI3K pathway enhances the differentiation of myofibroblasts, promotes the senescence, and inhibits the autophagy [55].

ECM receptors serve as the main mediator of myofibroblast differentiation. CD44, DDR1/2, and integrins were demonstrated as the most prominent ECM receptors of myofibroblasts [56, 57]. Primary ECM proteins of myofibroblasts contain collagen-I and collagen-III, but wound-repairing myofibroblasts also produce other types of components, including collagen-IV/V/VI, glycoproteins, and proteoglycans (e.g., fibronectin, laminin, and tenascin) [58-60]. Additionally, recent evidence suggests that CCN proteins, connective tissue growth factor (CTGF), and nephroblastoma are highly involved in the activation of myofibroblasts and tissue fibrosis [61]. Locally and serum upregulation of CTGF has been suggested to correlate with various fibrotic conditions. Mutations of the *CCN2* gene promoter are reported as a risk for systemic sclerosis in humans [62]. The CTGF monoclonal antibody, pamrevlumab, was approved by the Food and Drug Administration (FDA) for the treatment of IPF. However, activity of CCN2 occurs either up- or down-regulation of TGF-β signaling and then affects the effects of TGF-β on myofibroblast activation [58, 63]. In the model of folic-acid experimental nephropathy, CCN2 was shown to activate cellular senescence and lead to kidney fibrosis [64]. In senescent muscle stem cells (MuSCs), CCN2 was elevated during aging, and aerobic exercise could suppress the MuSCs-secretion of CCN2 [65]. In Duchenne muscular dystrophy (DMD), CCN2 appears to be involved in multiple processes of tissue fibrosis, including the inflammatory response, myofibroblast activation, and altered necrotic-regenerative foci [66]. In summary, the persistent activation of myofibroblasts in aging tissue contributes to fibrosis and a series of tissue dysfunctions. These dysregulations, including chronic inflammation, disruption of tissue architecture, and impaired regeneration, ultimately increase the tissue's susceptibility to disease and injury, which further enhances the differentiation of myofibroblasts.

In tumors, myofibroblasts play various roles, including the development of tumor stroma, tumor progression, invasion, and metastasis by promoting angiogenesis, mediating immune evasion, and reshaping a pro-tumorigenic environment. [67]. In tumor tissue, myCAFs are a subset of CAFs characterized by myofibroblast-like features. Herein, tumor myofibroblasts share the mechanisms of CAFs in contributing to tumor development, such as promoting physical barriers, influencing cancer cell growth, and affecting immune responses [68]. A subpopulation of senescent myofibroblasts or senCAFs was identified in mouse and human pancreatic ductal adenocarcinoma (PDAC). This group of senCAFs are located near tumor ducts and accumulated during the progression of PDAC [69]. This also indicates that the pathological feature of the senCAFs may be a potential prognostic biomarker for tumor therapy.

### 2.5 Adipocytes

Adipose tissue fibrosis is primarily caused by a disrupted metabolic balance between caloric intake and energy expenditure [70]. Obesity is the major driver of fibrosis in not only adipose tissue but also other organ dysfunctions [71]. In different organs, fibrosis in adipose tissue is a complex program involving multiple cell types, including adipocytes, mast cells, macrophages, and vascular endothelial cells [71]. Collagens are the major component of EMC in adipose tissue [72]. Obesity-associated adipocyte hypertrophy and hyperplasia cause tissue mass overgrowth and trigger inflammation [73]. This unique alteration of the tissue expansion results in the alteration of mechanical stability and elasticity [74]. Additionally, ECM-mediated signaling, including CD36, CD44, and integrins, provides the necessary cues to regulate cell behavior. It was indicated that MMP14 performs as the predominant collagenase and is required to promote the formation of white adipose tissue [75]. Senescent adipocytes were characterized by a remarkable increase in pro-inflammatory cytokines and ECM components [76]. During aging, adipocytes gradually stop dividing but keep the activation of metabolism, the accumulation of which in tissue cells promotes the excessive production of ECM and leads to organ fibrosis. It was demonstrated that senescent adipocytes also contribute to fibrosis in skeletal muscle. A type of mesenchymal stromal cell, named as fibro/adaptogenic progenitors (FAPs) can differentiate into both adipocytes and fibroblasts [77]. Senescent FAPs can promote muscle fibrosis and disturb the function of muscle stem cells (MuSCs), thereby impairing muscle regeneration [78].

Apart from directly producing ECM, adipocytes also produce metabolic factors and contribute to organ fibrosis by interacting with other cell types. It was reported that visceral adipose tissue promotes cardiac aging by promoting fibroblast senescence via osteopontin production [79]. Hypoxic adipose tissue is commonly considered a consequence of aging or obesity [80, 81]. Hypoxic adipocytes exhibit the expression of HIF1α, which subsequently induces alteration in the transcriptional profile engaging in ECM synthesis [72]. Fibrosis in obese fat tissue could be effectively prohibited by a chemical HIFα inhibitor or the overexpression of a dominant-negative mutant [82]. Cell death of adipocytes causes the remnant large lipid droplets to remain in the tissue, affecting the surrounding cells, including the macrophages, neutrophils, lymphocytes, and mast cells [83]. Together, adipocytes or their progenitors promote the aging-associated fibrosis via a complex cross-organ metabolic modulation and/or cell-cell interactions within adipose tissue.

Obesity-associated structural remodeling of the adipose tissue leads to the development of localized fibrosis. Moreover, tumor cells feed on the adipose tissue, resulting in an increased energetic supply to cancer cells, including the release of fatty acids and the enhanced production of lactate. [84]. Obesity-induced senescent adipocytes could create a fibrotic and inflamed TME to facilitate the progression of cancer [70]. The SASPs from senescent adipocytes promote the deposition of ECM components, alter the physical properties, and create a cancer-aggressive environment [85].

### 2.6 Macrophages

In contrast to directly producing ECM, macrophages exert their pro-fibrotic functions primarily through interactions with other cells. In response to tissue injury, macrophages act as the key immune regulators that promote both repair and the resolution of fibrosis. Given that chronic inflammation is commonly accompanied by tissue degeneration, it is noteworthy that inflammatory cells and mediators play critical roles in the development of fibrosis in aging [86-88]. During tissue aging and degeneration, macrophages undergo functional alterations that contribute to the persistence and progression of fibrosis [89]. Tissue-resident macrophages are capable of self-renewal, which enables them to maintain tissue homeostasis [90]. This suggests that macrophages maintain tissue homeostasis independently of the circulatory system. Under inflammatory conditions, macrophages derived from monocytes have been reported to regulate the inflammatory response and tissue repair [91]. Organ-specific macrophages, such as cardiac macrophages in the heart, Kupffer cells in the liver, renal macrophages in the kidney, and alveolar macrophages in the lung, exhibit distinct phenotypic characteristics and transcriptional profiles [92].

M1 macrophages are induced in response to Th1-related cytokines or bacterial lipopolysaccharide (LPS) stimulation. In contrast, M2 macrophages are induced by Th2 cytokines and other types of inflammatory cytokines [93]. In response to inflammation or injury, macrophages can polarize into M1 (pro-inflammatory) and M2 (anti-inflammatory/pro-repair) phenotypes, in which M2 plays a crucial role in tissue repair and contributes to fibrosis with prolonged activation [94]. Tissue stress causes an increase in M1 macrophages from local recruitment of monocytes, and the accumulation of M2 macrophages promotes the differentiation of myofibroblasts via the secretion of TGF-β, TNF-α, IL-1β, and IL-10, etc. [95, 96]. Herein, persistent activation of pro-reparative M2 macrophages contributes to the chronic fibrosis [1]. Aging increased the number of senescent macrophages, which characterize the altered morphology and secretion of SASP. Macrophage-derived SASP, which includes cytokines and chemokines, further stimulates myofibroblast differentiation and production of ECM [97]. In renal aging, for instance, tubular epithelial-mesenchymal transition and subsequent fibrosis were induced by SASP-related factors from senescent macrophages, including IL-6, TNF-α, and MCP-1 [98]. Obesity induced metabolic dysfunctions cause the SASP of macrophages and further promote the fibrotic program via triggering ECM production from mesenchymal stromal cells and preadipocytes [70].

Senescent macrophages contribute to cancer progression via SASPs. In the context of cancer, Senescent CAFs alter macrophage phenotypes towards a pro-tumorigenic state, leading to an immunosuppressive manner [69]. In a healthy context, macrophages are crucial for clearing excess ECM deposition. In contrast, the ability of macrophages to perform this function can be compromised in the chronic inflammation of tumors, leading to pathological fibrosis and tumor development [99]. It was reported that tumor-associated macrophages (TAM) could drive the overproduction of ECM proteins and result in tumor fibrosis [100]. Apart from stimulating the fibroblast by secreting profibrotic mediators, such as TGF-β and PDGF, macrophages can be recruited by fibroblast-derived factors like CSF1 and CCL2, which form a reinforcing loop for tumor fibrosis [101].

Taken together, fibrosis provides the main support for the growth, metastasis, and therapeutic resistance of the cancer cells. Similar to the imbalanced tissue regeneration, tumor tissue exhibits heterogeneity in cell diversity, particularly for CAFs. Aging results in the accumulation of senescent cells, thereby accelerating the progression of fibrosis, which further alters the components of the tumor microenvironment (TME) (Fig. 2). Therefore, anti-aging treatment for senescent cells may not only promote tissue regeneration.

**Figure 2.**
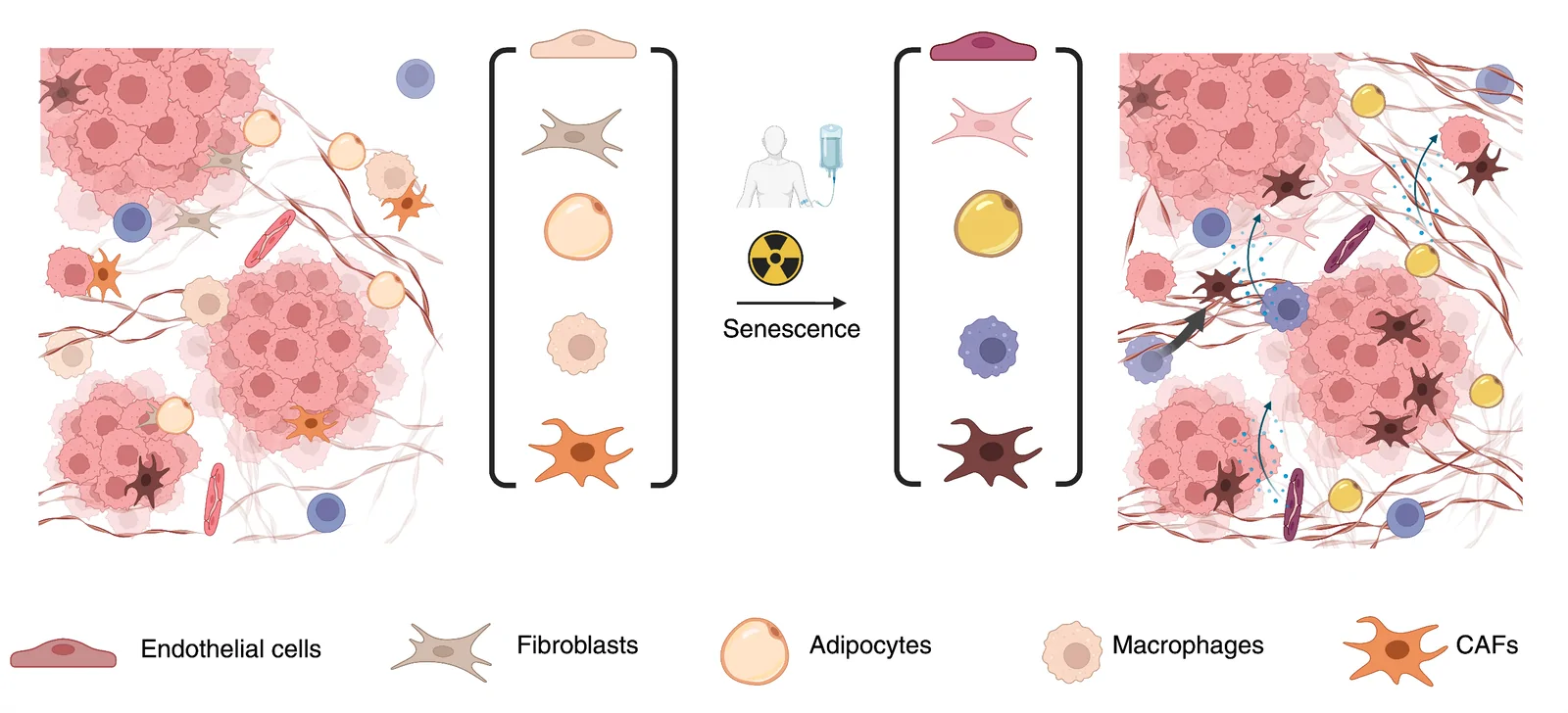
**Cell senescence and tumor fibrosis**. Chemo/radiation therapy-induced senescence promotes various cell feature alterations, thereby promoting the fibrosis of the tumor. These alterations include the transition of cell types into the characteristics of the CAFs, or an increase in the SASP, or an increase in the production of ECM components. The tumor fibrosis increased the stiffness of the tumor tissue, making it more difficult for the drug to reach the cancer cells. (This diagram was created with BioRender: https://www.biorender.com/)

## 3. Development of anti-fibrotic therapy

The FDA has approved two anti-fibrotic drugs for IPF. Nintedanib targets the tyrosine kinases PDGF receptor, FGF receptor, and VEGF receptor [102]. Although the mechanism is now incompletely understood, pirfenidone contributes to the suppression of multiple anti-inflammatory and anti-fibrotic effects [103]. The imbalance of regeneration capacity and hyperactivity of ECM production contributes to the aging-associated tissue degeneration. On the other hand, anti-fibrosis therapy would diminish the therapeutic resistance of the tumor. Most therapeutic candidates for anti-fibrosis remain in clinical trial stages. Due to limited efficacy or significant adverse effects, many of these candidates have failed to reach market approval. In the context of tissue degenerative changes, fibrosis is increasingly regarded as a biological marker rather than the sole or primary causal factor. Given the multiple types of cells involved in the fibrosis process, systemic therapy to normalize the scarring process, along with the inflammatory response and tissue regeneration, is key to longevity, rather than targeting fibrosis alone.

### 3.1 Dual-Action of anti-Aging and anti-Fibrotic

It is controversial whether fibrosis is a cause or a consequence of tissue aging. However, anti-aging therapy targeting organ fibrosis has shown its advantage in reducing organ dysfunctions. Targeting the aging mediators could exert the anti-fibrotic effects in a specific organ. Screening of compounds based on the synthesis of human collagen-I indicated that vitamin C, undecylenic acid, conjugated linoleic acid, glycolic acid, and citric acid are helpful for anti-fibrosis and premature aging [104]. Anti-aging strategies for muscle often include combating fibrosis, which has been employed as a novel therapeutic method for muscular diseases. Metformin, a well-established anti-aging drug, has demonstrated therapeutic effects in multiple organ fibrosis, including pulmonary fibrosis [105], liver fibrosis [106], and renal fibrosis [107]. Rapamycin, another well-known agent with anti-aging properties, has been shown to reduce collagen deposition, suppress myofibroblast activation, and attenuate inflammation during the progression of fibrosis [108-110]. From the metabolic regulating angle, dysfunction of mitochondria plays a significant role in aging and the development of fibrosis. The dysfunction of mitochondria involving release of mitochondrial DNA (mtDNA), oxidative stress, and mitochondrial autophagy (mitophagy) is closely connected to fibrotic disorders [111]. The compounds targeting mitochondrial oxidative stress, such as mitoquinone (MitoQ), have shown significant therapeutic effects in mitigating pulmonary fibrosis [112]. Additionally, NAD+ dependent proteins, such as Sirtuins (Sirt), which are widely recognized for their positive effects on age-related diseases, are also involved in regulating IPF [113]. Overexpression of sirtuin 3 (SIRT3), which is downregulated in the lungs of humans with IPF, could restore fibrosis resolution, correlating with the activation of pro-resolution macrophage functions [114]. Moreover, inhibition of fibroblast senescence via hirudin could result in the upregulation of the PGC-1α/SIRT3 pathway [115].

### 3.2 TCM for anti-fibrosis

TCM, characterized by its multidimensional, multi-targeted, and systematic approach, has demonstrated a promising advantage in anti-aging and fibrosis [116]. Until recently, variant plant-derived compounds have demonstrated regulatory effects in anti-fibrosis and anti-aging, including the regulation of liver inflammation, oxidative stress, the synthesis and secretion of fibrosis-related factors, as well as the activation, proliferation, and extracellular matrix synthesis of hepatic stellate cells [117]. A multiscale drug screening on cardiac fibroblasts indicated that artesunate, targeting myeloid differentiation factor 2 (MD2) and Toll-like receptor 4 (TLR4) signaling pathway, could alleviate heart fibrosis gene expression [118]. Noted, MD2/TLR4 plays a critical role in inflammatory response and various age-related diseases, particularly dilated cardiomyopathy [119]. Considering the shared molecular targets involved in tissue fibrosis and cell senescence, drug development targeting effects against fibrosis and aging hallmarks may be a promising future direction for exploring anti-fibrosis and anti-aging reagents.

Classic TCM composite formulae, such as *Yinchenhao Tang, Xiao Chaihu Tang, Buzhong Yiqi Tang*, and *Renshen Yangrong Tang*, have demonstrated efficacy in anti-fibrotic therapy [120-123]. Given the logic of TCM diagnosis and the multiple compound effects of TCM formulae, the medication would not only aim to inhibit excessive ECM production but also seek to regulate tissue homeostasis, including promoting regeneration, anti-inflammatory effects, and antioxidative stress. For instance, *Buzhong Yiqi Tang* and *Renshen Yangrong Tang have been reported to have therapeutic effects on liver fibrosis, as well as* immunomodulatory effects [122]. Moreover, convergent evidence indicated regenerative promotion effects of Renshen extract, the main component in *Renshen Yangrong Tang* [124-126]. To summarize, the combination therapy of anti-inflammation, regeneration promotion, and antioxidant effects of TCM supports tissue homeostasis, thereby switching the fibrosis pathology into a normalized tissue repair.

Based on the traditional Chinese medical principle, the occurrence of cancer is mainly due to an imbalance of Yin and Yang, which results in the mass or tumor [127]. The synergistic effects of TCM could enhance the effectiveness and reduce the side effects of conventional therapies, such as immunotherapy [128]. Bioactive components from TCMs have shown their capacity to modulate TME. Astragaloside IV could reduce the ability of CAFs to promote cancer spread by downregulating HOXA6 and ZBTB12 [129]. Phillygenin has been indicated to inhibit the pro-inflammatory cytokines, e.g., IL-6 and TNF-α, that CAFs produce, thereby affecting the immunosuppression of hepatocellular carcinoma [130]. Studies have shown that Yanggna Jiedu Sanjie and the alkaloid berberine can suppress the transition of epithelial cells to the mesenchymal lineage [131]. The active ingredients of TCMs and natural products can also suppress SASPs. Curcumin, a polyphenolic molecule extracted from *Curcuma longa*, has been shown to exert the effects of antioxidants, anti-inflammatory, anti-cancer, and anti-aging properties [132].

### 3.3 Advanced therapy in anti-fibrotic drug development

Gene and cell therapy has made significant advances in reducing off-target effects, achieving sustainable results, and promoting personalized medicine. It provides an innovative medical approach with the potential to target and cure organ fibrosis (Figure 3). For gene therapy, introducing genes that encode the proteins to inhibit the production of fibrotic factors or promote the ECM degradation is the main strategy. Current studies have demonstrated that restoring SERCA2a expression (a protein crucial for calcium regulation) via AAV1-derived gene delivery can reverse pulmonary fibrosis. The study indicated that a single administration of AAV1. SERCA2a gene therapy significantly reduced the lung fibrosis and was associated with vascular remodeling in the bleomycin-induced pulmonary fibrosis mouse model [133].

Oligonucleotide drugs, which are composed of short, designed DNA or RNA sequences, could target and modulate the expression of pro-fibrotic genes, such as TGF-β. Conjugated with lung-specific β-defensin 23, an antisense oligonucleotide targeting TGF-β mRNA showed promising treatment effects for pulmonary fibrosis with minimal side effects on other organs [134]. Moreover, small interfering (si) RNA targeting TGF-β mRNA can inhibit protein expression and attenuate hepatic fibrosis in a high-fat diet and carbon tetrachloride (CCl_4_)-induced rat model [135]. Compared with TGF-β siRNA, Xue et al. suggested that metalloproteinase (TIMP)-1 and TIMP-2 siRNAs provided the improved anti-fibrotic effects in the hepatic fibrosis model by exhibiting significantly increased expression levels of IFN-γ, but lower expression levels of IL-4 and IL-13 compared with the high-fat diet and CCl_4_-induced model group [136]. Apart from siRNA and ASO, aptamers, a short sequence of nucleic acid that binds to specific protein domains, are expected to treat tissue fibrosis [137]. In contrast to regulating mRNAs, aptamers bind to specific protein domains, resulting in improved precision regulation of downstream signaling. The aptamer-based drug 'Macugen', for treating neovascular age-related macular degeneration, was approved by the FDA in 2004, and a series of aptamer-based medications are in clinical pipelines [138]. CTGF protein has four domains that mediate a variety of signaling pathways, including those involving growth factors, integrins, and ECM proteins. The precision binding of the CTGF domain with aptamers is expected to treat fibrosis and other diseases, such as cancer and inflammatory disorders [139]. Delivery of mRNAs for gain-of-function therapy can obtain the anti-aging effects, thereby reaching the goal of anti-fibrosis. Recent research indicated that partial reprogramming, a new aging rejuvenating method with Yamanaka factors, remarkably reduced “mesenchymal drift”, which is considered an altered composition of stromal cell populations associated with fibrosis [140]. Unlike complete reprogramming for stem cell production, partial reprogramming focuses on the transient induction of pluripotency-associated factors, typically the Yamanaka factors (OCT4, SOX2, KLF4, and c-MYC, collectively known as OSKM), to reverse cellular aging phenotypes. This so-called rejuvenation therapy has been demonstrated to enhance regeneration, decrease fibrosis and improve tissue functional recovery in several tissues [141-144].

Cell therapies are getting increased attention for anti-aging and anti-fibrosis treatment. Chimeric antigen receptor T-cell (CAR-T) therapy, primarily demonstrated to be effective in hematological cancers, is now considered a novel treatment method for fibrosis. CAR-T cells can be infused and circulate to the heart, target the myofibroblasts that express FAP, and induce cytotoxicity to prevent the progression of fibrosis. For clinical effects, after 2 years of treatment with CD19 CAR-T cells combined with nintedanib, a case of systemic sclerosis-associated pulmonary fibrosis showed a 55% reduction in lung fibrosis [145]. In the chronic kidney disease mouse model, CAR-T therapy, which combines both adoptive transfer and CD5-lipid nanoparticle (LNP)-mediated *in vivo* generation, can significantly reduce fibrosis-induced pathologies, including kidney, myocardial interstitial, and perivascular fibrosis, with no evidence of notable toxicity [146]. In addition to CAR-T therapy, which targets and eliminates specific cell types, general cell therapies, particularly mesenchymal stem/stromal cells (MSCs), may also help exert anti-fibrotic effects and promote tissue regeneration through multiple mechanisms. It has been reported that MSCs can suppress fibrosis via the CTGF/FAK pathway in systemic lupus erythematosus [147]. In bleomycin-induced pulmonary fibrosis, combined treatment with pirfenidone, which targets TGF-β, and umbilical cord-derived mesenchymal stem cells (UC-MSCs) has recently demonstrated significantly improved pulmonary function, histopathology, and inflammatory factors (LNF-γ, IL-6, and TGF-β) [148]. Taken together, alternative medications such as TCM drugs, along with new CAR-T methods, pave a new way for antifibrosis treatment. However, the ongoing refinement and updating of antifibrotic targets are essential prerequisites for discovering and developing novel, effective therapies for organ fibrosis.

There are also limitations for CAR-T therapies, especially for anti-fibrosis. First is the risk of off-target cytotoxicity and tissue injury. Unlike tumors with well-defined neoantigens, fibrotic lesions lack unique and uniformly expressed targets [149]. Secondly, the microenvironment of fibrotic tissue is typically hypoxic, immunosuppressive, and densely collagenous, which limits the infiltration, persistence, and effector function of T cells. The in vivo delivery of CAR mRNA with nanoparticles could help overcome this limitation [150]. Most importantly, the antigen heterogeneity among multiple phenotypes (myofibroblast, senescent, and inflammatory) reduces the stability of antigen expression, leading to escape mechanisms and drug resistance. From an ethical perspective, the primary challenge of CAR-T therapy lies in balancing the therapeutic benefits and safety risks. From an economic perspective, both CAR-T and gene therapies entail high production and implementation costs due to the requirement for high-grade vector production under strict Good Manufacturing Practice conditions. The need for personalized processing, long-term follow-up, and specialized infrastructure further adds to the overall cost burden.

**Figure 3.**
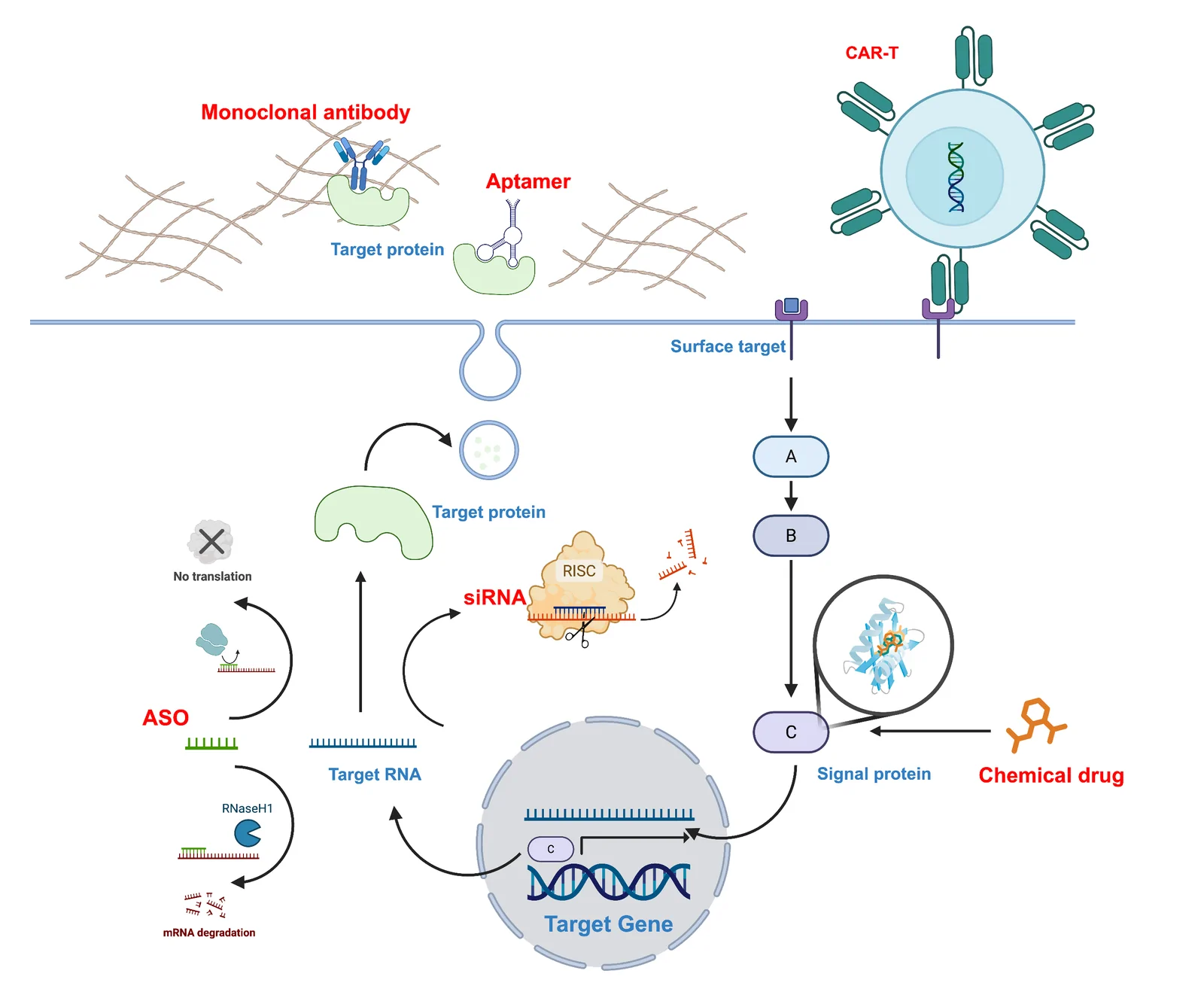
**Recent advances for anti-fibrotic therapy**. Potential targets and mechanisms of the different anti-fibrotic therapies, including ASO, siRNA, chemical drugs, aptamers, monoclonal antibodies, and CAR-T. (This diagram was created with BioRender: https://www.biorender.com/)

## 4. Innovative methods for anti-fibrosis investigation

Drug re-identification may produce unexpected effects on complex aging-related disorders, such as metabolic diseases, cancer, neurodegeneration, and organ fibrosis. New drug delivery systems, like nanomaterials, could enhance the effectiveness of existing drugs by modifying tissue distribution and improving cellular targeting. Although drug development for antifibrotic therapy is increasing, it remains largely empirical and high-risk without a robust and predictive disease model. Recent disease models derived from patients' iPSCs, or tissue provide a humanized analysis tool to help re-identify drugs for antifibrosis diseases.

### 4.1 Nano-medications for antifibrosis and longevity

During fibrosis, excessive ECM deposition leads to increased mechanical stiffness and alters the cell environment. This tighter mesh in stiffened tissue and the dense ECM barrier blocks the permeability of the interstitial space, physically blocking molecule diffusion [151]. Additionally, fibrosis-induced tissue stiffness causes reduced vascular perfusion and higher interstitial fluid pressure, ultimately affecting cell uptake and causing uneven drug distribution *in situ* [152, 153]. Nanotechnology-based drug delivery systems can enhance the solubility and bioavailability of natural compounds, monoclonal antibodies, and chemical drugs [154]. Loading nanoparticles improves drug targeting to specific profibrotic signaling pathways, reduces oxidative stress, modulates the immune response, and scavenges senescent cells, thereby enhancing the effectiveness of anti-fibrosis and anti-aging therapies. For example, Prussian blue (PB) nanozymes have a well-defined crystalline structure and possess multifunctional enzymatic activities, including peroxidase-, superoxide dismutase-, and catalase-like functions, along with improved hydroxyl radical scavenging efficiency. These PB nanozymes show potential in treating fibrotic liver disease by scavenging reactive oxygen species (ROS), modulating the immune response, and promoting hepatic stellate cell quiescence [155]. Moreover, mesoporous silica nanoparticles loaded with doxorubicin can induce anti-aging effects, eliminate senescent cells, and reduce pulmonary fibrosis [156].

During disease development, nanoparticles could target specific cell types by affinity to the physicochemical environmental specificities, such as pH, temperature, or specific molecules [157]. He et al. discovered that in chronic kidney disease-associated fibrosis, biomimetic high-density lipoprotein (bHDL) lipid nanoparticles presented the promising targeting capacity to injured tubular epithelial cells mediated by kidney injury molecule-1(KIM-1) [158]. For diminishing the senescent cells, the remarkable development of nanomaterials for their unique optical, magnetic, and electrical properties, such as accumulating of p53, p16, and p21, as well as neighboring cells with secretion of IL-6 and IL-8 [159]. Experiments have suggested that the nanocarrier preferentially releases the drug molecule in patients with congenital keratinization disorder and human senescent fibroblasts [160], indicating the effectiveness of the nanoparticle delivery system in anti-fibrosis and anti-aging.

Nanomedicine provides the solution to delivering therapeutic agents under dense ECM in tumor tissue. Using physical/chemical or biological approaches, nanomedicine could eliminate or degrade the dense ECM and reduce tumor stiffness to improve drug penetration [161]. Besides treatment of cancer cells, researchers are increasingly focusing on targeting CAFs for stromal-rich tumor therapies. The advances to harness nanotechnologies in cancer treatment are mainly based on the capacity of nanoparticles to deliver the therapeutic agents directly to CAFs and reduce the tumor stiffness [162]. Wang et al conjugated the doxorubicin (DOX)-loaded hydroxyethyl starch-IR780 nanoparticles (NPs) with Cys-Arg-Glu-Lys-Ala (CREKA) peptide, which presented a special affinity for fibronectin overexpressed on CAFs and significant inhibition in 4T1 tumor [163]. Liu et al developed the CAF inhibitor-loaded nanomedicine glutathione-responsive nanocomplex (GNC) could suppress CAFs and increase the therapeutic effects of immunogenic chemotherapy, indicating the overcoming of chemoresistance [164]. Targeting the TME hypoxia, Zou et al designed a pH/redox dual-responsive nanomedicine for remodeling of CAFs and prolonging the amplification of oxidative stress [165].

### 4.2 New disease model for antifibrosis

A non-animal humanized disease model is a new direction for drug discovery. Organoids derived from human induced pluripotent stem cells (iPSCs) or human tissue offer 3-dimensional and self-organizing tissue structures, providing a unique advantage in replicating physiological relevance, cell-cell interactions, and cell-ECM interactions with the complete patient's genetic background [166]. Organoids derived from iPSCs have been applied in duplicating tissue degenerations, e.g., amyotrophic lateral sclerosis (ALS), DMD, and Parkinson's disease [167-169]. On the other hand, organoids derived from patients' tissue, such as tumor organoids, could provide an individual disease model for clinical drug testing, such as CAR-T therapy [170]. The importance of organoids as an alternative research tool has gotten increasing attention. Until recently, the National Institute of Health (NIH) established the first national organoids development center to reduce the reliance on animal models. 2D cultured fibroblasts derived from iPSCs have been used for high-throughput screening of drugs against fibrosis, such as cardiac-specific fibroblasts [118]. Single-cell sequencing analysis provides a powerful tool for identifying the presence of fibroblasts in tissue-specific organoids. To date, emerging evidence suggests that tissue-derived organoids may serve as a potential model for organ-specific fibrosis. Lung organoids derived from native lung tissue can be self-organized and used to evaluate the efficacy of anti-fibrotic drugs based on the readout of α-SMA staining and single-cell sequencing [171]. Wang et al. developed a native tissue-derived lung organoid-based fibrotic model, established through fibroblast coculture [171]. Dowbaj et al. developed the mouse-derived multicellular organoids system composed of adult hepatocytes, cholangiocytes, and mesenchymal cells for duplicating the architecture of the liver periportal region. This model could be further assembled with cholangiocytes and portal fibroblasts to duplicate cholestatic disease and biliary fibrosis [172]. Tumor organoids can also replicate peritumoral fibrosis in solid cancers. Developed by Jang et al., multi-layer spheroids formed by fibrosis-encapsulated tumoroids established a stable fibrotic-like phenotype for up to 14 days, which enables an applicable tool for studying the role of fibrosis in the therapeutic resistance of solid cancers [173].

Organoids derived from iPSCs could provide an *in vitro* disease model for diseases that are difficult to biopsy, such as cardiovascular or neurological diseases. Song et al. reported that self-organizing heart organoids from human iPSCs contain cardiomyocytes, fibroblasts, and endothelial cells. This heart organoid could replicate ischemia-reperfusion-induced cardiac fibrosis, characterized by collagen deposition, disrupted calcium ion handling, and electrophysiological anomalies [174]. Using 11 different healthy and diseased iPSCs, Ouchi et al. developed human liver organoids that were composed of hepatocytes, stellate cells, and Kupffer-like cells. Moreover, these liver organoids exhibited free acid-induced tissue stiffness, as detected by atomic force microscopy [175]. Engineered homozygous mutation of *PKDH^M36^* iPSC-derived multi-linage hepatic organoids presented the Autosomal Recessive Polycystic Kidney Disease (ARPKD) associated liver fibrosis, as indicated by Masson staining and collagen fiber analysis [176]. Organoids can also be generated as a disease model for intestinal disease-associated fibrosis. The intestinal organoids from the iPSCs from patients with Crohn’s disease showed TGFβ-induced fibrosis, as reflected by the elevated expression of *Col1a1, Fibronectin*, *TIMP1* gene, and epithelial-mesenchymal transition [177].

**Figure 4.**
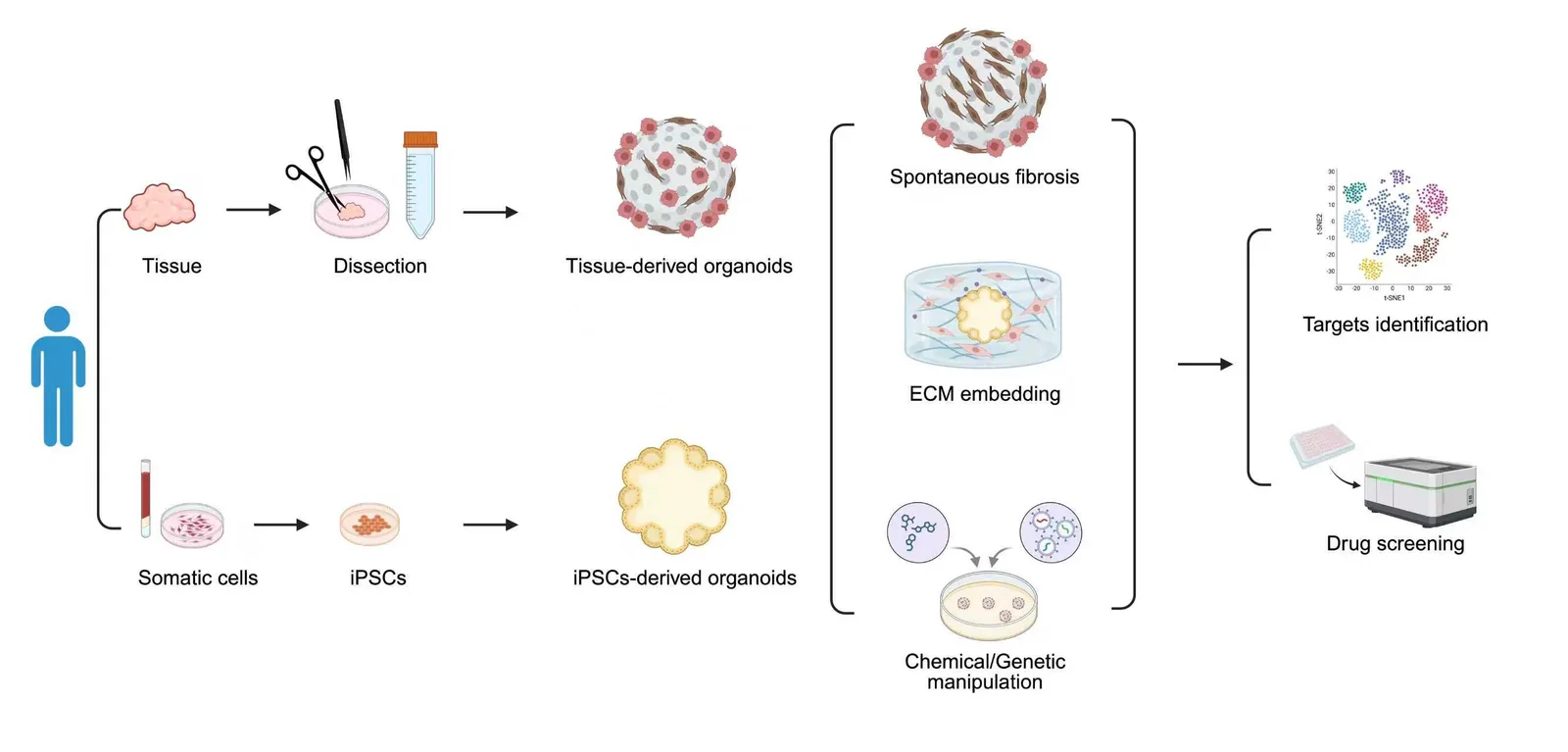
**Modeling fibrosis using human-derived organoids**. Schematic progress of establishment of tissue-/iPSCs- derived organoids and the application details for anti-fibrotic drug development. (This diagram was created with BioRender: https://www.biorender.com/)

Tissue-specific organoids could duplicate fibrosis induced by niches regulating and multi-cellular signaling. For cancer investigation, organoids could be raised from the tumor biopsy. Patient-specific tumor organoids offer advancements in biobanking, providing insights into the genetic, proteomic, morphological, and pharmacotypic features of the tumor [178]. Tumor organoids can mimic the TME that includes neoplastic and non-neoplastic components. Single-cell RNA sequencing revealed that glioblastoma organoids comprise a diverse range of non-neoplastic cell types, including macrophages, microglia, and T cells [179]. On the other hand, incorporating CAFs via co-culture could create the TME *in vitro* and enhance the stability of the organoids in duplicating parental tumor tissue [180]. It was reported that in the presence of co-cultural CAFs or the conditional medium treatment, tumor organoids from liver cancer conferred resistance to anticancer medications, including sorafenib, regorafenib, and 5-fluorouracil [181].

The hallmarks of aging, including DNA damage, glycation, oxidation, and protein misfolding, could be induced in organoids via chemical or genetic manipulation. Both tissue- or iPSCs-derived organoids could provide a promising platform for investigating the pathophysiology of aging-related diseases. Spontaneous accumulation of amyloid β was observed in organoids derived from iPSCs or neural spheroids that transdifferentiate from the fibroblasts of AD patients [182, 183]. Fibroblast-derived skin organoids exposed to mitomycin C can acquire characteristics of old skin, including decreased filaggrin expression, dysregulated epidermal differentiation, reduced elastin, and collagen [184]. Figure 4 presents the application of human-derived organoids from tissue or iPSCs to replicate the pathologies of fibrosis (Figure 4). By combining advanced technology, 3D printing organoid chips can mimic aging-associated systemic cross-organ mechanisms, including fibrosis.

## 5. Conclusion and perspective

Aging leads to organ dysfunctions *via* multiple cellular events that promote fibrosis, including diminished capacity for normal tissue regeneration, increased inflammatory responses, and a transient fibroblast lineage. Organ fibrosis caused by chronic conditions can be seen as an imbalance of tissue scarring, involving impaired tissue recovery and excessive ECM production. As a hallmark of aging, fibrosis accelerates tissue dysfunction and induces various processes such as alterations in cell diversity, changes in the mechanotransduction environment, and inflammatory responses. Antifibrotic therapy may restore tissue architecture and delay cellular decline, offering anti-aging benefits. Developing drugs to target profibrotic factors could help achieve anti-aging effects. Besides, anti-aging agents that focus on immune modulation, tissue repair, and antioxidant effects also display antifibrotic properties. Fibrosis also serves as a key feature of the TME, providing crucial support to tumor growth and metastasis. Aging, chemotherapy, and radiotherapy that induce cell senescence would promote the cell death of the cancer cells, but may also trigger the non-neoplastic cells, such as CAFs. SASPs play a significant role in remodeling the TME and promote tumor fibrosis. Herein, clearing the senescent non-neoplastic pro-fibrotic cells in tumor tissue would provide a synergistic therapeutic effect.

Therapeutic targets for anti-aging and anti-fibrosis may benefit from the overlap in their mechanisms. Multiple omics technologies can reveal the temporal and spatial molecular networks involved in the development of fibrosis. A database from the aging-associated organ cell atlas could provide deep insights into the possible mediators and the key cell types that contribute to tissue fibrosis. Additionally, CRISPR screening, which uses CRISPR/Cas9 technology to disrupt genes in cells, can identify novel targets for anti-fibrotic therapies through loss-of-function screening for hits [185]. These technologies, combined with artificial intelligence (AI), offer a breakthrough in creating an AI virtual cell (AIVC), a multi-scale, multi-modal, large-neural-network-based model that can represent and simulate the behavior of molecules, cells, and tissues across various states [186]. In the future, omics data from the new disease model via organoids may provide more information to decode the mechanism of fibrosis. AI virtual cells involving fibroblasts or those related to fibrosis progression may be utilized to explore new targets or drugs for anti-aging and anti-fibrosis therapies.
